# Assessing Baccalaureate Nursing Students’ Antibiotic Stewardship Knowledge Using Virtual Standardized Patient Simulations

**DOI:** 10.1017/ash.2021.68

**Published:** 2021-07-29

**Authors:** Mary Lou Manning, Monika Pogorzelska-Maziarz, David Jack, Lori Wheeler

## Abstract

**Background:** According to the Centers for Disease Control and Prevention, the single most important factor leading to the development of antibiotic resistance (AMR) is the use of antibiotics. Studies indicate that up to 50% of hospitalized patients receive at least 1 antibiotic, half of which are inappropriate. The outpatient setting accounts for >60% of antibiotic use and over half of these prescriptions are inappropriate. Antibiotic stewardship programs improve appropriate antibiotic use, reduce AMR, decrease complications of antibiotic use, and improve patient outcomes. Building a nursing workforce with necessary AMR and antibiotic stewardship knowledge and skill is critical. Nursing graduates can translate knowledge into practice, promoting the judicious use of antibiotics to keep patients safe from antibiotic harm. **Methods:** Third-year baccalaureate nursing students enrolled in a fall 2020 health promotion course at an urban university affiliated with an academic medical center participated. Students received a 3-hour lecture on antibiotics, AMR and antibiotic stewardship nursing practices and actively engaged in antibiotic stewardship simulations using standardized patient (SP) encounters. The SP participants were specifically trained for these activities. Simulations included a 30-minute brief before and a 60-minute briefing after the activities. All activities occurred via video conferencing. Case scenarios, developed by the authors, focused on penicillin-allergy delabeling of an adolescent prior to elective surgery and appropriate use of antibiotics in managing pediatric urinary tract infections and acute otitis media (AOM). Before-and-after tests were used to assess the impact on AMR and antibiotic stewardship knowledge. **Results:** Over a period of 4 days, all enrolled students (n = 165) participated in 1 three-hour virtual simulation session. Using Zoom video conferencing with multiple breakout rooms, the activities were easily managed. During the simulations, students often struggled with reading an antibiogram and applying the concept of “watchful waiting” in AOM management. Significant differences were found in before-and-after test results, with significant improvement in students’ general and specific knowledge and awareness of antibiotics (*P* < .01). During the debriefing sessions, students reported increased awareness related to their role in advancing the judicious use of antibiotics. **Conclusions:** Initially, we planned to conduct in-person SP simulations. Due to the COVID-19 pandemic, faculty and students demonstrated remarkable flexibility and resilience as we successfully converted to a virtual format. Virtual lecture and SP simulations, followed by debriefing, was an effective approach to educate baccalaureate nursing students about AMR and their role in antibiotic stewardship. Areas for course content improvement were identified.

**Funding:** No

**Disclosures:** None

